# Tropical oils consumption and health: a scoping review to inform the development of guidelines in tropical regions

**DOI:** 10.1186/s12889-024-19949-x

**Published:** 2024-09-10

**Authors:** Thomas Hormenu, Iddrisu Salifu, Juliet Elikem Paku, Peace Yaa Kordowu, Adams Abdul-Karim, Thomas Boateng Gyan, Immanuel Asiedu, Osman Abdul-Ganiyu, Mustapha Amoadu

**Affiliations:** 1 Department of Health, Physical Education and Recreation, University of Cape Coast, Cape Coast, Ghana; 2 Cardiometabolic Epidemiology Research Laboratory, University of Cape Coast, Cape Coast, Ghana

**Keywords:** Tropical oils, Palm oil, Coconut oil, Health benefits, Consumption guidelines

## Abstract

**Background:**

Tropical oils such as palm and coconut oils are renowned for their high saturated fat content and culinary versatility. However, their consumption has sparked debate regarding their health benefits and production concerns. The purpose of this review was to map existing evidence on the health benefits and challenges associated with the consumption of tropical oils.

**Method:**

The recommendations for conducting a scoping review by Arksey and O’Malley were followed. PubMed, Dimensions AI, Central, JSTOR Google, Google Scholar, and ProQuest databases were searched for relevant papers. The predetermined keywords used were Consumption” AND “Tropical oil,” as well as “Health benefits” OR “Health challenges” AND “Tropical Countries.” Peer-reviewed and grey literature published in English were eligible for this review.

**Result:**

Tropical oils, such as palm and coconut oils, provide health benefits including essential vitamins (A and E) that enhance ocular health, boost immunity, and support growth. They are also recognised for their role in managing high blood sugar, obesity, and cholesterol levels, while offering antioxidant and anti-inflammatory properties. These oils have wound-healing abilities and are commonly used in infant nutrition and traditional cooking. Nevertheless, prolonged and repeated use of tropical oils to high temperature can degrade vitamin E, whereas excessive intake may result in overdose. Health concerns include oxidative risks, diabetes, cancer, coronary heart disease, high blood pressure, and acrylamide formation due to production challenges excessive consumption. Additional issues include obesity, suboptimal oil production, misconceptions, regulatory obstacles, and preferences for alternative fats.

**Conclusion:**

This review suggest that tropical oils provide essential health benefits, including vitamins and antioxidant properties, but pose significant health risks and production challenges, particularly when exposed to high temperatures and through excessive intake. Guidelines on the consumption of tropical oils in the tropical regions are necessary to regulate their consumption.

**Supplementary Information:**

The online version contains supplementary material available at 10.1186/s12889-024-19949-x.

## Introduction

Tropical oils, such as palm oil and coconut oil, have deep-rooted historical and cultural significance in tropical regions, which serve as integral components of local cuisines in Africa [1, 2]. These oils, extracted from palm fruits and coconuts, are celebrated for their unique qualities, notably, their high saturated fat content and versatility in cooking [3, 4]. Nevertheless, their consumption has ignited extensive debates, warranting careful examination of their potential health benefits and ongoing concerns regarding their production [5].

Palm oil, which is widely employed in the food industry, cosmetics, and various industrial applications, holds a prominent place in the tropical regions due to its economic importance. It contains a blend of saturated and unsaturated fats, which makes it a suitable choice for various cooking methods and food products [6]. However, discussions regarding the health implications of palm oil consumption persist [7]. The high saturated fat content of palm oil has raised concerns similar to those associated with coconut oil, prompting ongoing scrutiny regarding its potential health effects [3]. This ongoing debate emphasises the economic benefits of palm oil and the need for closer examination of its impact on health.

Coconut oil praised for its diverse applications in cooking, cosmetics, and traditional medicine, is particularly favored in tropical regions because of its unique medium-chain fatty acids, which are believed to provide rapid energy and metabolic support [8, 9]. However, debates surrounding the health implications of coconut oil consumption, primarily linked to its high saturated fat content, have contributed to the ongoing discourse on this topic [10, 11].

The justification for conducting this scoping review is rooted in the multifaceted nature of palm and coconut oil consumption in tropical regions, with a primary focus on health considerations. It is imperative to assess the health implications of consuming tropical oils, notably coconut and palm oils, as they have been associated with various health outcomes [10, 11]. While there are debates regarding the potential health benefits of coconut oil, concerns have arisen over the high saturated fat content of palm oil and its potential negative impact on cardiovascular health [3, 6, 12]. Evidence shows that there are both positive and negative health outcomes associated with these oils. For instance, some studies suggest that the medium-chain fatty acids in coconut oil may aid in weight loss and improve metabolic parameters [8, 9]. Conversely, other reviews have raised concerns about the high saturated fat content in coconut and palm oils, linking them to increased cardiovascular risks [10, 11]. Instructively, a search through literature revealed lack of guidelines on the consumption of these oils, their health benefits and health risks associated with repeated usage.

Therefore, this review aims to provide a balanced assessment of the existing evidence concerning the health outcomes associated with the consumption of these oils. This review acknowledges the deep-rooted dietary traditions and cultural significance of palm and coconut oils in the tropical regions. These oils are integral to the cultural and culinary heritage of these regions, and this review respects and preserves this heritage while simultaneously addressing pertinent health concerns [13, 14]. The benefits of this review include informing the development of guidelines for the consumption of palm and coconut oils in tropical regions. By offering a holistic view of the health benefits and challenges associated with these oils, this study supports informed decision-making and policy development, effectively balancing the preservation of cultural heritage with the promotion of public health.

## Methods

The scoping review followed the methodological framework established by Arksey and O’Malley [15]. This framework encompasses five key stages: formulating the research questions; identifying relevant studies; selecting the studies; organizing and summarizing the data; and compiling, condensing, and presenting the findings [15]. The primary research questions driving this scoping review are as follows: (1) What are the health advantages associated with tropical oil consumption? and (2) What are the health challenges associated with the consumption of tropical oil? An extensive search strategy was to identify pertinent studies. The search was conducted using four primary electronic databases: PubMed, Dimensions AI, Central, and JSTOR. In addition, supplementary searches for grey literature were carried out on Google, Google Scholar, and ProQuest. The search terms employed included various combinations of “Consumption” AND “Tropical oil,” as well as “Health benefits” OR “Health challenges” AND “Tropical Countries.” Boolean operators (AND, OR) were employed to effectively merge these search terms. MeSH terms were devised for use in the PubMed search and subsequently adjusted for application in other databases. The MeSH terms used in the PubMed search are listed in Table 1.

To ensure the selection of studies appropriate for this review, we established clear inclusion and exclusion criteria. After conducting searches in various databases, we imported the retrieved records into reference management software called Mendeley, where we systematically removed duplicate entries. The next step involved a thorough review of the titles and abstracts of the records. This initial screening process was carried out by a team of 15 graduate students and teaching assistants who had received training for this purpose. The entire process was supervised by the authors (MA and TH).

**Table 1:** Search strategy in PubMed

Search (#)	Search terms
1	“Consumption” OR “Intake” OR “Use” OR “Absorption” OR “Eating”
2	“Tropical Oil” OR “Copra Oil” OR “Cocos Nucifera Oil” OR “Coconut Butter” OR “Cococin” OR “Dietary Fats, Unsaturated” OR “Plant Oils” OR “Cocos” OR “Caprylates” OR “Palm Oil” OR “Elaeis Guineensis Oil” OR “African Oil Palm Oil” OR “Elaeis Oleifera Oil” OR “American Oil Palm Oil” OR “Vegetable Oil” OR “Tropical Oil”.
3	“Health benefits” OR “Health advantages” OR “Medical benefits” OR “Therapeutic effects” OR “Clinical advantages”
4	“Health challenges” OR “Health issues” OR “Health problems” OR “Medical challenges” OR “Health difficulties”
5	“Tropical region” OR “Afghanistan” OR “Algeria” OR “Angola” OR “Antigua and Barbuda” OR “Argentina” OR “Australia” OR “The Bahamas” OR “Bangladesh” OR “Barbados” OR “Belize” OR “Benin” OR “Bhutan” OR “Bolivia” OR “Botswana” OR “Brazil” OR “Brunei” OR “Burkina Faso” OR “Burundi” OR “Cambodia” OR “Cameroon” OR “Cape Verde” OR “Central African Republic” OR “Chad” OR “Colombia” OR “Comoros” OR “Congo, Dr Congo” OR “Congo Republic” OR “Costa Rica” OR “Cuba” OR “Djibouti” OR “Dominica” OR “Dominican Republic” OR “East Timor” OR “Ecuador” OR “Egypt” OR “El Salvador” OR “Equatorial Guinea” OR “Eritrea” OR “Eswatini” OR “Ethiopia” OR “Fiji” OR “Gabon” OR “Gambia” OR “Ghana” OR “Grenada” OR “Guatemala” OR “Guinea” OR “Guinea-Bissau” OR “Guyana” OR “Haiti” OR “Honduras” OR “India” OR “Indonesia” OR “Iran” OR “Iraq” OR “Ivory Coast” OR “Jamaica” OR “Jordan” OR “Kenya” OR “Kiribati” OR “Kuwait” OR “Laos” OR “Lebanon” OR “Lesotho” OR “Liberia” OR “Libya” OR “Madagascar” OR “Malawi” OR “Malaysia” OR “Maldives” OR “Mali” OR “Marshall Islands” OR “Mauritania” OR “Mauritius” OR “Mexico” OR “Micronesia” OR “Mozambique” OR “Myanmar” OR “Namibia” OR “Nauru” OR “Nepal” OR “Nicaragua” OR “Niger” OR “Nigeria” OR “Oman” OR “Pakistan” OR “Palau” OR “Panama” OR “Papua New Guinea” OR “Paraguay” OR “Peru” OR “Philippines” OR “Qatar” OR “Rwanda” OR “Saint Kitts and Nevis” OR “Saint Lucia” OR “Saint Vincent and the Grenadines” OR “Samoa” OR “Sao Tome and Principe” OR “Saudi Arabia” OR “Senegal” OR “Seychelles” OR “Sierra Leone” OR “Singapore” OR “Solomon Islands” OR “Somalia” OR “South Africa” OR “South Sudan” OR “Sri Lanka” OR “Sudan” OR “Suriname” OR “Syria” OR “Tanzania” OR “Thailand” OR “Togo” OR “Tonga” OR “Trinidad and Tobago” OR “Tuvalu” OR “Uganda” OR “United Arab Emirates” OR “Uruguay” OR “Vanuatu” OR “Venezuela” OR “Vietnam” OR “Yemen” OR “Zambia” OR “Zimbabwe”
6	#1 AND #2 AND #3 AND 5 Not Animal* Filters activated (01/01/2015 to 26/10/2023), English Language
7	#1 AND #2 AND #4 AND 5 Not Animal* Filters activated (01/01/2015 to 26/10/2023), English Language

Subsequently, full-text versions of the records that met the eligibility criteria based on their abstracts and titles were obtained for further evaluation. In this phase, the authors screened the full-text records, making decisions regarding inclusion and exclusion. Records that were excluded during this stage were documented, along with the reasons for their exclusion. The eligibility criteria used for this screening process are in Table 2.

**Table 2:** Eligibility criteria

Inclusion criteria	Exclusion criteria
• A study was conducted on the health benefits and challenges of tropical oils (Palm oil, coconut, palm kernel oil, sunflower oil, soybean oil, groundnut oil) consumption.	• Studies conducted outside the Tropical Countries.
• Studies that utilise primary data and reviews	• Studies that did not report variables of interest.
• Studies published in the English language	• Studies published in language other than English.
• Peer-reviewed and grey literature.	• Conference papers, abstracts, letters, editorials, preprints and commentaries.
• Studies published on from 01/01/2015 to 26/10/2023	• Studies published before 2015.

Data charting involved the creation of a structured form to systematically extract pertinent information from selected studies. The data extraction process encompassed various aspects such as study characteristics (e.g., author, year), study design, population, sample size, and health benefits and challenges of tropical oil consumption. To ensure accuracy and reliability, the data charting process was divided into two distinct groups consisting of three independent researchers. This division of labour was implemented to guarantee the precision of data extraction. In cases in which discrepancies or disagreements arose between the two groups, these issues were addressed and resolved during regular meetings with the authors.

Upon completion of data charting, the extracted data were subjected to a synthesis process aimed at providing an overview the health benefits and challenges associated with tropical oils consumption. The scoping review used qualitative methods to present the results. Thematic analysis was employed to identify the common benefits and challenges of consuming tropical oils that emerged across the included studies. Furthermore, narrative synthesis was undertaken to offer insights into the findings and trends observed in the literature. Throughout the review process, consultations were sought to enhance the rigor and replicability of this study. A chartered librarian at Sam Jonah Library, Kwame Kodua-Ntim, was consulted during the search and paper screening phases to ensure the thoroughness of the search process. Additionally, review and subject matter experts were engaged to ensure that the review process adhered to the scoping review approach, thus enhancing the overall quality and robustness of the study.

### Ethical consideration

Ethical approval and consent was not sought for the present study since the study files used in this study are published and available in the public domain. Some of the studies that support the findings of this review but are restricted to the public domain were sorted upon reasonable request. Table S1 presents details on extracted data.

## Results

### Search results

**Fig. 1:**
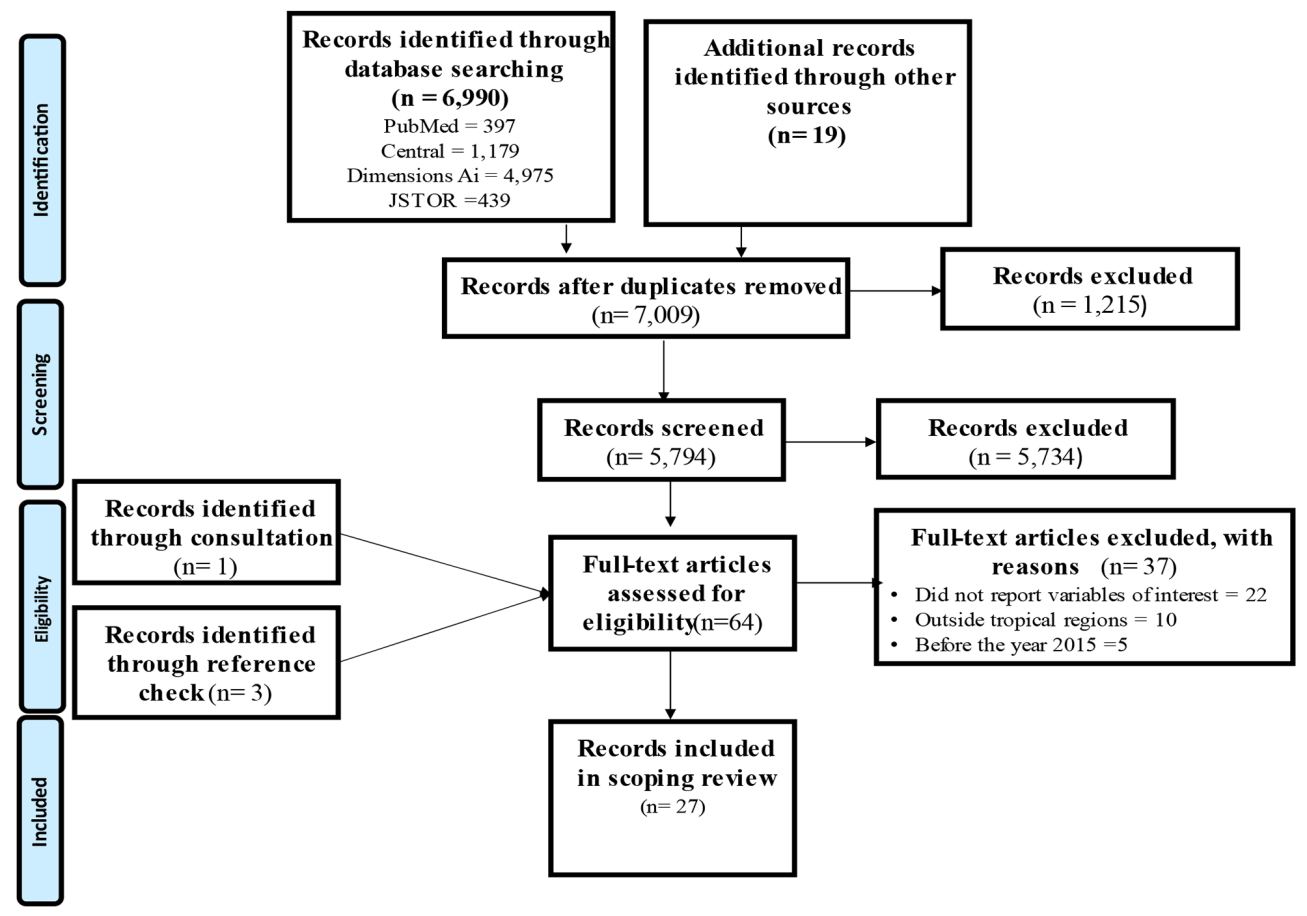
PRISMA flow chart for the search results and screening process

Search conducted in the four main databases produced 6,990 records, while an additional search in other databases produced 19 records. Therefore, 7,009 records were retrieved. In the first level of screening, 1,215 duplicate records were removed using Mendeley software. Furthermore, 5,794 records were screened, and 5,734 records were removed. Records removed at this phase were abstracts and records that were of no interest to the subject explored. In the final screening, 64 full-text records were screened against the eligibility criteria. The 64 records included one that was obtained through consultation with the chartered librarian and three records through reference checking of the eligible full-text records. Twenty-seven records were finally included in the review. See Fig. 1 for more details on the search results and the screening process.

### Study characteristics

**Fig. 2:**
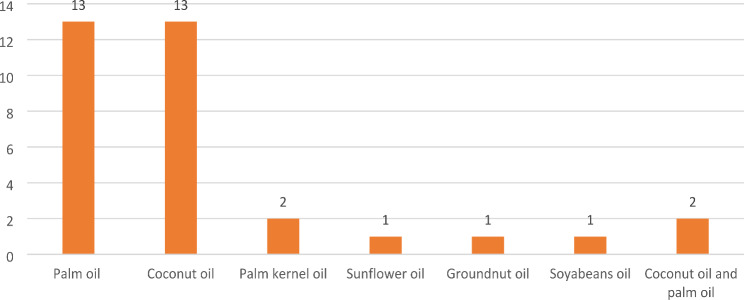
Type of tropical oil reviewed studies explored

**Fig. 3:**
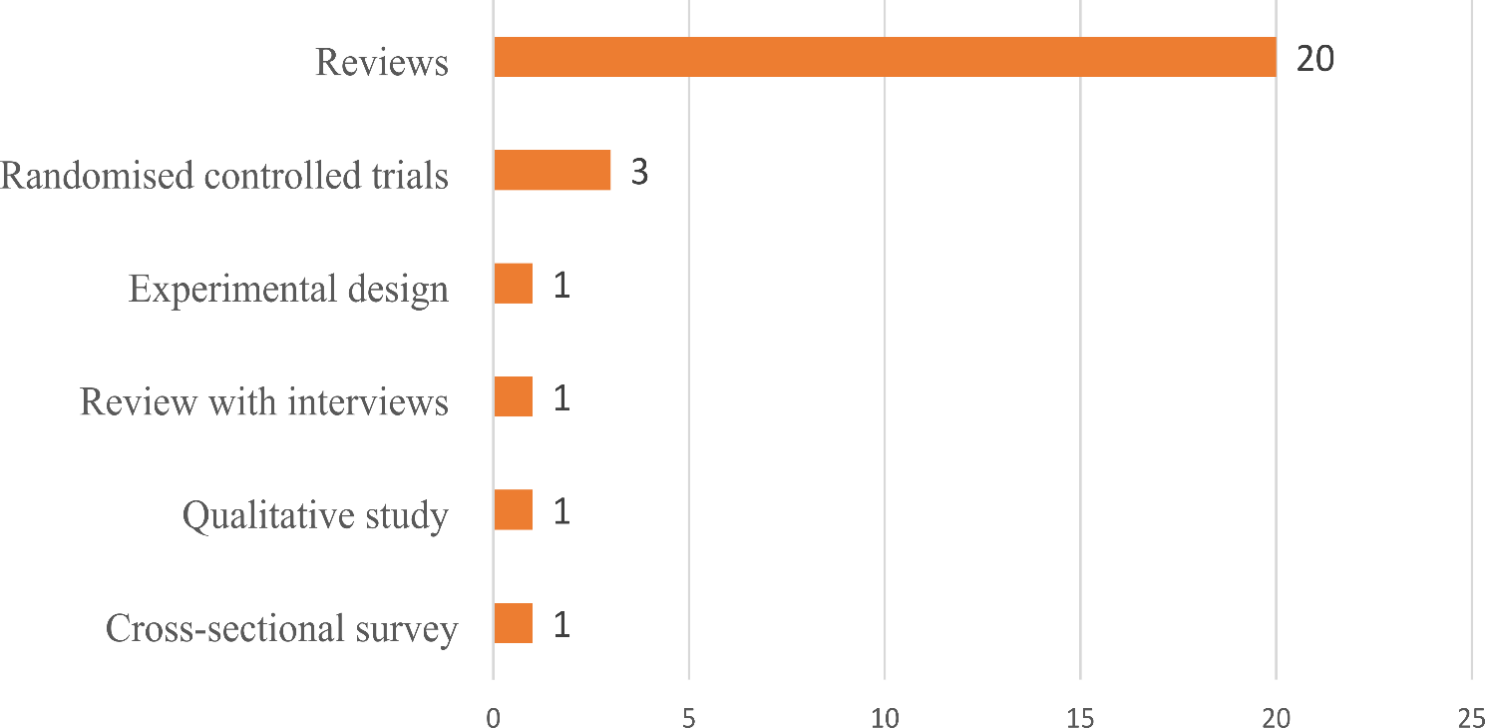
Study designs explored by include studies

**Fig. 4:**
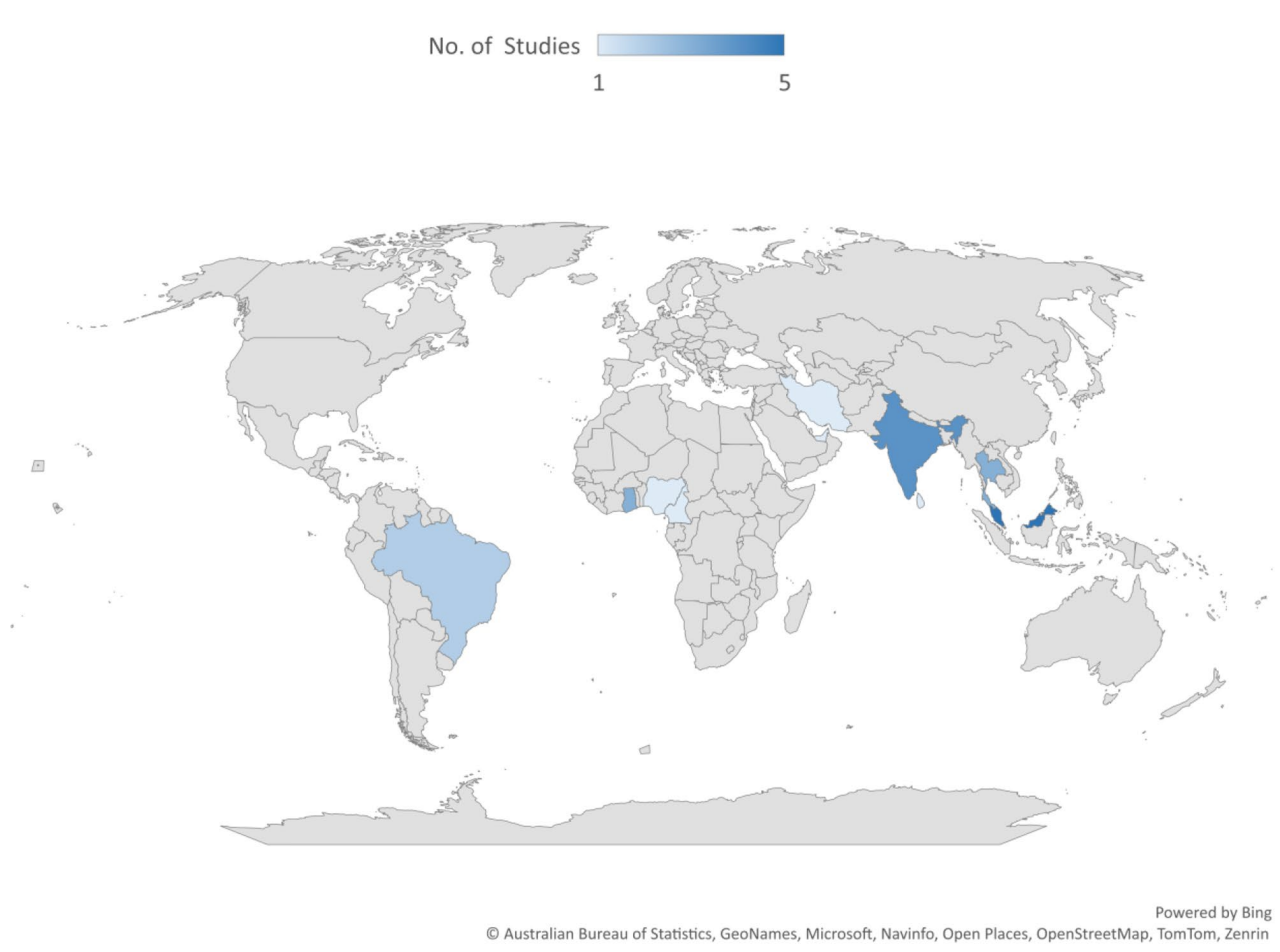
Map showing countries included studies were condcute

The tropical oils most explored among the included studies were palm (13 studies) and coconut oil (13 studies). The details of tropical oil explored in the included studies are shown in Fig. 2. However, one includes study captured other tropical oils, such as palm kernel and groundnut oil. Most studies (21 studies) were document reviews and evidence synthesis (See Fig. 3 for details). The majority (five) of the included studies were conducted in Malaysia (See Fig. 4 for details). Table S1 presents the extracted data.

### Benefits of tropical oil

This section provides a comprehensive overview of the benefits associated with tropical oil consumption, with specific focus on palm and coconut oils. The review encompasses two themes, including nutritional and pharnacological benefits. These categories were used to enhance evidence synthesis. The details are presented in Table 3.

**Table 3:** Benefits of tropical oils

General Themes	Specific Theme	Author
Nutritional benefits	Palm oil contains Vitamins A	Mba et al., 2015; MacAurthur et al., 2021; Mba et al.,2015; Hanafah et al., 2021; Kappally et al., 2015; Tan et al., 2021; Koushki et al., 2015
	Palm oil contain vitamin which aids in ocular rewetting	Kappally et al., 2015
	Palm oil contains Vitamins E	Mba et al., 2015; MacAurthur et al., 2021; Mba et al., 2015; Hanafiah et al., 2022; Boateng et al., 2016
	Palm oil provides nutrients for growth	Abdullahi et al., 2023; Nondzor et al., 2015; Boateng et al., 2016
	Coconut oil provides nutrients for growth	Konar et al., 2020
	Palm oil is used in preparing infant milk	Hasanuzzaman & Nahar, 2022; Suryani et al. 2020; Konar et al., 2020
	Processed food	Shankar et al., 2017
	Palm oil is used as the main traditional cooking oil.	Ntsefong et al., 2016
Pharmacological benefits	Palm oil reduces high blood sugar	Hasanuzzaman & Nahar, 2022; Suryani et al. 2020; IMOISI et al., 2015;
	Coconut oil and soyabeans oil were effective in management of obesity.	Vogel, et al., 2020
	Coconut oil improves cognitive function and strengthens immunity	Joshi et al., 2020; Kappally et al., 2015
	Palm oil and coconut oil reduces cholesterol level	Jayawardena et al., 2021; Boateng et al., 2016; Joshi et al., 2020; Tan et al., 2021; Kappally et al., 2015; Narayanankutty et al., 2018; Imoisi et al., 2015; Neelakantan et al., 2020; Naidu and Moorthy, 2021; Unhapipatpong et al., 2021;
	Coconut oil helps in prevention of dental caries and atopic dermatitis and hair damage	Chew, 2018; Santos et al.,2019; Naidu and Moorthy, 2021
	Palm oil consumption reduce the risk of cancer.	Deen et al., 2021; Koushki et al., 2015; Tan et al., 2021; Kappally et al., 2015
	Virgin Coconut oil improves lipid profile	Kappally et al., 2015
	Palm oil consumption increases energy level	Shankar et al., 2017
	Palm oil helps in reducing coronary heart diseases	Joshi et al., 2020; Kappally et al., 2015; Narayanankutty et al., 2018; Unhapipatpong et al., 2021;
	Palm helps in reducing diabetes.	Vogel, et al., 2020; Santos et al.,2019; Narayanankutty et al., 2018; Neelakantan et al., 2020; Naidu and Moorthy, 2021
	Palm oil aid in treatment of bones	Santos et al.,2019; Naidu and Moorthy, 2021
	Palm oil acts as anti-inflammatory agent	Chew, 2018; Deen et al., 2021; Kappally et al., 2015; Narayanankutty et al., 2018; Ayanlowo et al., 2022; Neelakantan et al., 2019; Naidu and Moorthy, 2021
	palm oil has anti-aging agent	Ayanlowo et al., 2022;
	Anti-oxidant	Joshi et al., 2020; Deen et al., 2021; Koushki et al., 2015; Tan et al., 2021; Narayanankutty et al., 2018; Ayanlowo et al., 2022; Suryani et al.,2020;
	Palm kernel consumption promotes neuromotor development	Jayawardena et al., 2021;
	Palm kernel softens skins	Ayanlowo et al., 2022;
	coconut oil regulates insulate resistance	Narayanankutty et al., 2018
	Coconut oil restores liver functioning	Narayanankutty et al., 2018
	Palm oil reduces weight and body fatness	Suryani et al., 2020; Neelakantan et al.,2019; Ayanlowo et al., 2022; Kappally et al., 2015; Vogel, et al., 2020; Konar et al., 2020
	Palm kernel consumption leads to reasonable weight gain	Ayanlowo et al., 2022
	Palm oil aids in wound healing	Suryani et al., 2020; Ayanlowo et al., 2022; Jayawardena et al., 2021; YikLing Chew, 2019

### Nutritional benefits

Tropical oils, particularly palm oil, are valuable sources of essential vitamins. Palm oil contains Vitamin A, which aids in ocular rewetting and thus contributes to eye health [16]. In addition, it is rich in Vitamin E, which plays a crucial role in overall health [17–19]. Palm oil is also used in the preparation of infant milk, signifying its importance in providing essential nutrition to infants [20, 21, 29]. Both palm and coconut oils are rich sources of nutrients that are necessary for growth [19, 29, 31, 32]. Palm oil has traditionally been used as the primary cooking oil in many regions, highlighting its cultural and culinary significance [33]. Tropical oils play a significant role in processed food production, with palm oil being utilized in various food products [30].

### Pharmacological benefits

Parmacological benefits of tropical oils were categorised into pre-clinical and clinical benefits. This was done to enshance synthesis and understanding.

### Preclinical benfits

Consumption of palm oil is correlated with reductions in weight and body fat, while palm kernel oil consumption can lead to reasonable weight gain [9, 16, 21, 26, 28, 29]. Additionally, tropical oils, particularly palm oil, have wound-healing properties, making them valuable in medical contexts in tropical regions [21, 24–26]. Both palm and coconut oils exhibit anti-inflammatory, antioxidant, and anti-aging properties, further enhancing their therapeutic potential [12, 16, 25, 26].

### Clinical benefits

Palm oil has been found to reduce high blood sugar levels and lower the risk of diabetes and other non-communicable diseases [20–22]. Coconut oil is effective for the management of obesity [9], and it is also known to improve cognitive function and strengthen immunity [16, 23]. Both palm and coconut oils have been linked to a reduction in cholesterol levels, aiding in the prevention of coronary heart diseases [19, 24]. In particular, palm oil is associated with a lower risk of cancer [12]. Additionally, palm kernel oil consumption is associated with neuromotor development [24] and skin softening [26]. Coconut oil regulates insulin resistance and restores liver function [27].

### Health safety Assessment of the consumption of tropical oils

This section presents the health challenges and issues associated with the consumption of tropical oils. The review is organised around several key themes, each of which is supported by relevant citations to provide a comprehensive understanding of the concerns associated with these tropical oils. The challenges associated with the consumption of tropical oils are reported in Table 4.

**Table 4:** Health challenges / issues of tropical oils

General themes	Specific theme	Author
Vitamins	Prolong and repeated exposure of palm oil to high temperatures degrade vitamin E.	Mba et al., 2015; Ma & Lee, 2016
	Vitamin E is loss during processing and refining.	Mba et al., 2015; Ma & Lee, 2016
	Overdose of vitamin E as a result of high amount of palm oil consumption	Mba et al., 2015; Ma & Lee, 2016
Clinical	Consumption of unrefined palm oil leads to risk of hydrolysis and oxidation.	MacAurthur et al., 2021
	Excessive consumption of Palm oil leads diabetes mellitus	Boateng et al., 2016
	Palm oil generate reactive oxygen that induce DNA damage	Tan et al., 2021
	Too much consumption of palm oil leads to certain types of cancer	Ntsefong et al., 2016;
	Excessive consumption of Palm oil leads to increased risk of developing coronary artery disease,	Ntsefong et al., 2016;
	Excessive consumption of high blood pressure,	Boateng et al., 2016
	Palm oil causes cancer due to formation of acrylamide at high frying temperatures.	Boateng et al., 2016
	The composition of fatty acid of palm oil is assumed to be a cause of coronary heart disease.	Koushki et al., 2015
	Consumption of palm oil containing Sudan IV dye causes cancer	MacAurthur et al., 2021
	Unrefined oils are high in cholesterol	Nondzor et al., 2015
Anthropometric	Excessive intake of palm and coconut is strongly associated with an increased prevalence of obesity.	Boateng et al., 2016
Quality	Compromise on materials leads to poor quality palm oils	MacAurthur et al., 2021; Hasanuzzaman & Nahar, 2022; Narayanankutty et al., 2018; Abdullahi et al., 2023
	Variation in processing of palm oil leads to Microbial contamination	MacAurthur et al., 2021; Hasanuzzaman & Nahar, 2022; Narayanankutty et al., 2018; Abdullahi et al., 2023
Misconception	Misconception that palm oil consumption leads to health challenges deter people from using it.	Shankar et al., 2017; Kappally et al., 2015
Regulations	Complex regulatory environment with little space for health-related considerations prevent people from palm oil consumption	Shankar et al., 2017
Preference	People prefer to use saturated fat to the palm oil	Unhapipatpong et al., 2021
Research	Lack of supportive scientific evidence deters people from coconut oil consumption.	Deen et al., 2021

### Impact on vitamins

Prolonged and repeated use of palm oil to high temperatures has been shown to degrade vitamin E, which can lead to potential nutritional deficiencies [17, 34]. Additionally, vitamin E is lost during the processing and refining of palm oil, diminishing its nutritional value [17, 34]. A high consumption of palm oil can result in an overdose of vitamin E, posing health risks [17, 34].

### Clinical health issues

The consumption of unrefined palm oil carries the risk of hydrolysis and oxidation, which canlead to health problems [18]. Excessive consumption of palm oil has been associated with an increased risk of diabetes mellitus [19]. Palm oil has also been shown to generate reactive oxygen species that can induce DNA damage, potentially contributing to various health issues [35]. Furthermore, excessive consumption of palm oil has been linked to certain types of cancer, coronary artery disease, high blood pressure, and the formation of acrylamide at high frying temperatures, further highlighting health concerns [19, 33, 36]. The fatty acid composition of palm oil is assumed to contribute to coronary heart disease [36], and palm oil contaminated with Sudan IV dye has been linked to cancer [18]. Unrefined oils, including palm oil, can be high in cholesterol, posing additional health risks [32]. Excessive intake of palm and coconut oils is strongly associated with an increased prevalence of obesity [19].

### Quality issues

Compromises in the materials used in palm oil production can lead to poor quality oils, impacting the overall safety and effectiveness of the product [20, 27, 31]. Variation in the processing of palm oil have been associated with microbial contamination, further affecting the quality of the oil [18].

### Misconceptions and regulatory hurdles

Misconceptions regarding the health challenges associated with palm oil consumption can deter people from using palm oil [16, 30]. Complex regulatory environments with limited consideration for health-related factors can also discourage palm oil consumption [30].

### Preference and research

Some individuals prefer saturated fats to palm oil [11]. Furthermore, the lack of supportive scientific evidence can deter people from consuming coconut oil [12].

## Discussions

### Summary of findings

Tropical oils, such as palm and coconut oil, offer various health benefits, including essential vitamins A and E, which promote ocular health, improve immunity, and support growth. They are known to reduce high blood sugar levels, manage obesity, and lower cholesterol levels. Additionally, these oils possess antioxidant and anti-inflammatory properties and wound-healing abilities and play a role in infant nutrition and traditional cooking. However, their consumption is not without a major health challenge. Prolonged use may inhibit vitamin E growth, leading to potential deficiencies, and a high intake can result in an overdose. Health concerns include oxidative risks, diabetes, cancer, coronary heart disease, high blood pressure, and acrylamide formation. Additional issues encompass obesity, low-quality oil production, misconceptions, regulatory hurdles, and preferences for alternative fats. This review provides a holistic view of the complex nature of tropical oils and balances their benefits and associated health considerations.

### Health benefits of consuming tropical oils

The presence of essential vitamins, notably Vitamin A and E, in tropical oils such as palm oil addresses the unique dietary challenges often encountered in tropical regions [16, 17]. These areas may face constraints in achieving dietary diversity, owning to environmental factors and agricultural limitations. Therefore, incorporation of such vitamins in the local diet is highly significant. By combatting micronutrient deficiencies, these oils contribute to enhanced eye health and overall well-being, effectively reducing the burden of nutrition-related health issues [16, 17].

Tropical oils, such as palm and coconut oils, exhibit various clinical benefits, including management of high blood sugar, obesity, and inflammation-related diseases [9, 12, 20]. In tropical regions, where the prevalence of NCDs such as diabetes and heart diseases are on the rise, these findings are of paramount importance. By offering natural solutions to mitigate high blood sugar levels and reduce obesity, these oils curb the NCDs burden [9, 20]. Additionally, their anti-inflammatory and antioxidant properties are promising for the prevention and management of various chronic diseases such as cancer [12]. This addresses a pressing health challenge and aligns with global health goals, including those outlined in the Sustainable Development Goals (SDGs).

The use of palm oil in processed food production provides an economic opportunity in tropical regions [30]. As the demand for processed foods continues to grow, it creates a potential revenue stream and job opportunities, thereby contributing to economic development. Furthermore, the traditional use of palm oil as a primary cooking oil in many regions has underscored its cultural importance [33]. Preserving these culinary traditions not only enhances cultural sustainability, but also has implications for overall well-being, as traditional diets often emphasize balanced and locally sourced foods, which can positively impact health outcomes [33].

### Health challenges associated with the use of tropical oils

The unique findings regarding the health challenges associated with tropical oils stem from a combination of factors, including the historical and entrenched use of palm oil in tropical diets [16, 33]. Prolonged consumption patterns in these regions have revealed both the beneficial and adverse effects of these oils. The high prevalence of non-communicable diseases (NCDs), such as diabetes, heart diseases, and obesity in tropical regions underlines the relevance of unique findings. These regions are in the midst of an epidemiological transition characterised by rising NCD rates [19], making dietary factors such as excessive tropical oil consumption a significant contributor to these health challenges. Additionally, variations in processing methods, quality control, and product purity play a pivotal role in the health impact of these oils [20, 27, 31]. Differences in the refinement and processing of oils can lead to varying health outcomes and quality control issues and possible contamination can further compound the health challenges associated with these oils [18]. The implications of the health challenges associated with tropical oils are multifaceted. They direct relevant for addressing the burden of non-communicable diseases, such as diabetes, heart diseases, and obesity, in tropical regions [19]. Furthermore, they have implications for achieving the United Nations’ (UN) Sustainable Development Goals (SDGs), particularly SDG 3 (Good Health and Well-being) and SDG 12 (Responsible Consumption and Production). These findings underscore the importance of promoting healthy diets and reducing NCD burden in the pursuit of SDGs. Healthcare systems in these regions face added pressure owning to the increased prevalence of NCDs, making it imperative to develop strategies that address both prevention and treatment [19]. Balancing economic development through palm oil production with the associated health challenges is a complex issue. Sustainable and responsible production methods can contribute to economic development, while mitigating health risks [20, 27, 31]. Finally, improving well-being in tropical regions necessitates a comprehensive approach that includes education and awareness campaigns to guide consumers toward healthier choices and to preserve cultural dietary traditions [16, 30].

### Guidelines for palm and coconut oils consumption in tropical regions

Encouraging moderation and balance is paramount in the consumption of palm and coconut oils in tropical regions. While these oils offer a range of health benefits, it is essential to advocate their moderate use in daily diets to avoid potential health challenges. Emphasising a balanced approach and diverse diet, where these oils are complemented with a variety of other foods, will help ensure individuals receive a broad spectrum of nutrients. Quality control in the production of these oils is critical. High-quality standards for processing and refining should be enforced to reduce health risks associated with contamination or oxidation.

Furthermore, education and awareness campaigns should be implemented to inform consumers about the advantages and risks associated with palm and coconut oils. Disseminating evidence-based information can guide consumers in making informed dietary choices, dispelling misconceptions that may deter or misguide consumption. Preserving traditional cooking practices incorporating palm oil is a valuable approach. These practices often involve the use of small quantities of palm oil in diverse locally sourced dishes, contributing to healthier dietary patterns. Additionally, integrating healthcare systems into these efforts, monitoring and regulating production, and promoting responsible consumption and production practices that align with the Sustainable Development Goals (SDG 12) will play a crucial role in addressing the complex nature of palm and coconut oil consumption in tropical regions. These guidelines aim to strike a balance between leveraging the health benefits of these oils and addressing the health challenges associated with their consumption, ultimately contributing to the overall well-being in tropical regions.

### Limitations in this review

This review used only studies published in English, which could reduce the volume of retrieved studies and the depth of evidence. In addition, evidence from this study was retrieved from tropical regions, which could also lead to a reduced volume and depth of evidence used for this synthesis. Most of the included studies were document reviews and evidence from various countries. Document review and existing evidence may be subject to selection bias, as the choice of which documents to review or which existing evidence to consider can be influenced by the researchers’ preferences or the availability of data. This bias can lead to an incomplete or skewed representation of the topic. The quality and currency of the data in documents and existing evidence can vary significantly. Some information may be outdated or may be based on less rigorous research methods, potentially affecting the accuracy of the findings. This can limit the depth of understanding of the factors and dynamics at play on the topic under investigation. Most of the included studies in this review are based on document reviews, which may lack the context and detailed insights that primary research methods, such as surveys or interviews, can offer. This limitation may restrict the depth of analysis and discussion. Regardless of these limitations, the researchers ensured robustness in search and screening procedures, extraction and collating extracted data and the findings, recommendations conclusion from this review were opted.

### Recommendation for future studies

Future research should conduct longitudinal studies in tropical regions to assess the long-term health effects of palm and coconut oil consumption. This would provide valuable insights into the cumulative impact of these oils on health outcomes, addressing the limitation of relying on existing evidence and document reviews. Future studies should investigate the relationship between dietary patterns, cultural influences, and t consumption of tropical oils. Understanding how cultural practices and food traditions intersect with oil consumption can help tailor dietary recommendations and public health interventions more effectively by considering cultural contexts. Finally, future studies should explore the interplay between sustainable palm and coconut oil production practices and their impact on health outcomes. Research should examine how responsible production methods, quality controls, and regulatory measures can mitigate health risks while contributing to economic development in tropical regions. One significant gap is the lack of longitudinal studies and randomized controlled trials that examine the long-term health effects of tropical oil consumption. Current evidence is often based on short-term studies, which may not fully capture the potential chronic health impacts, including the development of cardiovascular diseases, diabetes, and other non-communicable diseases. Besides, evidence on the health challenges associated with prolonged and excessive consumption of tropical oils is not conclusive. The degradation of essential vitamins, such as Vitamin E, due to high-temperature processing of palm oil, is an area that requires more in-depth research. Understanding the extent of nutrient loss and its implications for nutritional deficiencies could help in developing better processing methods to preserve these vital nutrients. More high-quality studies, such as randomised controlled trials and longitudinal designs, are needed to thoroughly explore these potential health risks.

## Conclusions

This comprehensive review highlights the multifaceted nature of tropical oils, specifically palm and coconut oils, in tropical regions. While these oils offer significant health benefits, such as essential vitamins, disease management, and cultural significance, they are not without health challenges, including the risk of nutrient imbalances and the exacerbation of non-communicable diseases. These findings emphasize the critical need for moderation, education, and quality control of the consumption and production of these oils. Moreover, the review underscores broader implications for public health, sustainable development, and well-being in tropical regions. Although, limitations exist, the robust search and screening procedures undertaken provide valuable insights, and the proposed guidelines aim to strike a balance between harnessing the benefits and mitigating the challenges associated with these tropical oils.

## Electronic supplementary material

Below is the link to the electronic supplementary material.

Supplementary Material 1

## Data Availability

No datasets were generated or analysed during the current study.
