# Supplementary material for: Tropical oils consumption and health: a scoping review to inform the development of guidelines in tropical regions
Source: BMC Public Health. 2024 Sep 10;24:2468. doi: 10.1186/s12889-024-19949-x (PMC11389204; doi:10.1186/s12889-024-19949-x)
Supplement: Supplementary file 1 — Supplementary Material 1 [file 12889_2024_19949_MOESM1_ESM.pdf]

Table S1: Data extracted from reviewed studies

Author and country	Purpose of the study	Design	Population	Sample size	Challenges/issues	Benefits
MacAurthur et al., 2021<br><br>Ghana	To evaluate the quality and safety of palm oil samples from seven major cities in Ghana using a completely randomized design with three replications	Randomized design	Ghana<br><br>Samples of palm oil	70	1. Risk of hydrolysis and oxidation as a result of consuming unrefined crude palm oil (CPO)<br><br>2. Consumption of palm oil containing Sudan IV dye which contains casinogenic effect<br><br>3. Longer length of time of storing fruits coupled with the use of inappropriate equipment and inadequate processing time compromise the quality of CPO.	1. Palm contains alpha-and beta carotenes, and lycopene, which account for its characteristic dark red colour<br>2. Carotene in palm oil has vitamin A and E to mitigate against vitamin A and E deficiency.
Hasanuzzaman & Nahar, 2022<br><br>Malasia	Aimed at utilization potentials of oilseeds in the tropics, therapeutic considerations, source of vital nutrients for consumers, prospects, and challenges of oilseed production for increased global competitiveness	review	Malaysia		1. Lack of modernization, low oil recoveries, lack of standardization of product quality, use of local technologies and Widespread inefficiency affects domestic markets and export quality.	1. Palm oil reduces high blood sugar and cholesterol level.<br>2. It is used as a component of infant milk powders because of its easy digestibility and stable flavor.<br>3. Palm oil has natural vitamin E which protects and repairs damaged cells.
Suryani et al., 2020<br><br>Thailand	To study the unique factors of virgin coconut oil (VCO) compared with coconut oil.	experimental	Malaysia			1. Virgin Coconut Oil is useful against microbes, bacteria and viruses.<br>2. Virgin Coconut Oil useful for helping one lose weight in terms of metabolism.<br>3. Virgin Coconut Oil causes people to consume less carbohydrates, which eventually reduces body weight.<br>4. Virgin Coconut Oil is used as antioxidants<br>5. Virgin Coconut Oil reduces high blood pressure and

						6. Virgin Coconut Oil affects the healing after an ovariectomy
Shankar et al<br><br>2017<br>Thailand	To understand the prospects for future health-focused policy development to limit food use of palm oil and promote a greater diversity of oils in Thailand's food system	Interviews	Thailand	18 stakeholders<br><br>8 from public sector<br><br>7 from private sector<br><br>3 from NGO	1. Doubt and misperception about negative health implications as a result of palm oil consumption.<br>2. Complex regulatory environment with little space for health-related considerations prevent people from palm oil consumption	1. Palm oil increases energy availability than any other food group.<br>2. Palm oil serves as an ingredient in a large variety of processed food product.
Mba et al, 2015<br><br>Nigeria	To combine and condense the body of research on the processing, characterization and use of palm oil especially in frying as well as suggest areas that need further research.	Review	Nigeria		1. There is loss of some vitamin Es during processing and refining of palm oil<br>2. Prolong use of Palm oil (vitamin E) inhibits growth.	1. Palm oil has a unique fatty acid and triacylglycerol profile which makes it suitable for numerous food applications<br>2. Palm oil is used to prepare infant formulae and provides vitamins A and E to the body.<br>3. The tocopherols and tocotrienols in the palm oil act as anti-cancer, ant- inflammatory agents and also control atherosclerosis, and decrease cholesterol.
Unhapipatpong et al, 2021<br><br>Thailand	To investigate the effect of tropical oil (palm and coconut oils), lard, and other common vegetable oils (soybean and rice bran oils) that are widely used in tropical and Asian countries on lipid profiles.	Review	Thailand		1. The crave for the consumption of saturated fat rich oil particularly from animal fats inhibit the use of palm oil.	1. Palm oil was associated with a significantly lower Lipoprotein cholesterol than those of other Saturated Facts.<br>2. Palm oil leads to reduction in coronary heart diseases
Hanafiah et al, 2022<br><br>Malaysia	To summarize evidence on the impact of Palm oil on health, social and economic aspects, environment, and biodiversity in the Malaysian context, and discuss mitigation strategies based on the sustainable development goals (SDGs)	Review			1. Excessive consumption of palm oil elevates the risk of cardiovascular diseases.	1. Palm oil provides phytonutrients<br>2. Palm oil is very rich in vitamin A and E
Naidu & Moorthy, 2021<br><br>Malaysia	To provide a critical review of the key sustainability issues faced by the Malaysian palm oil industry as the second biggest exporter of palm oil to the global market.	review			1. Negative perception of palm oil containing monochloropropane-1,2-diol (3-MCPD) and glycidyl esters (GE) which compromises the safety and quality	1. The nutraceuticals and phytonutrients from palm oil reduce cholesterol and diabetic levels in adults<br>2. palm oil tocotrienol as an antioxidant serves in prevention and treatment of bone-related illness.<br>3. The anti-inflammatory effect of palm oil phenolics may prevent the

					2. Excessive consumption of high amount of saturated fats of palm oil is link to heart disease.	formation of neurodegenerative diseases such as Alzheimer's and Parkinson's.
Neelakantan et al, 2020<br><br>Singapore	To assess the effect of coconut oil consumption on blood lipids and other cardiovascular risk factors compared with other cooking oils using data from clinical trials	Review			1. High Coconut oil consumption results in significantly higher Lopoprotein cholesterol and leads to cardiovascular diseases.	1. Consumption of coconut fat reduces serum cholesterol levels compared with non-tropical vegetable oils.<br>2. In addition to lipid concentrations, coconut oil has been suggested to alleviate inflammation,<br>3. coconut oil also improves glucose homeostasis.<br>4. Coconut oil reduce body fatness.
Imoisi et al,<br><br>2015<br><br>Nigeria	To determine the health and nutritional implication of palm oil.	Review			1. Oxidized palm oil induces an adverse effect on plasma lipid profile, free fatty acids, phospholipids and cerebrosides.<br>2. Oxidized palm oil induces reproductive toxicity and organ toxicity particularly of the kidneys, lungs, liver and heart.	1. Palm oil has high solid-glyceride content, giving the required consistency without hydrogenation.<br>2., Palm oil is very resistant to oxidation and therefore has a long shelf life<br>3. Palm oil leads to reduction in the risk of arterial thrombosis and atherosclerosis, inhibition of cholesterol biosynthesis and platelet aggregation,<br>4. Palm oil reduction in blood pressure.
Ayanlowo et al<br><br>2022<br><br>Nigeria	To ascertain the traditional, medical, cosmetics, and other usage of oils for skin and scalp care in the Africa.	Review and<br><br>interviews	Traditional and alternate healers	15	1. Palm Kernel Oil has been associated with an increase risk of coagulase negative staphylococci infections in preterm neonates; miliaria and folliculitis in babies.	1. Coconut oil possesses anti-inflammatory, antioxidant and antiaging effects<br>2. Topical applications of virgin coconut oil are effective in promoting wound healing<br>3. Coconut oil enhances protective barrier function of the stratum corneum by increasing the expression of cornified envelope components.<br>4. Palm kernel oil reduces trans epidermal water loss and increase skin capacitance, which are beneficial and effective in conditions such as xerosis, atopic dermatitis.<br>5. Palm kernel oil is an effective emollient: It softens the skin, improves

						skin barrier function and reduces trans-epidermal water loss, and<br>6. Palm kernel oil improves thermoregulation<br>7. Palm kernel oil leads to reasonable weight gain, better sleep-wake pattern, and enhanced neuromotor development.
Narayanankutty et al<br><br>2018<br><br>India	This review emphasizes the use of Virgin Cooking Oil in the prevention and amelioration of several degenerative diseases, including cardiovascular disease and cancers.	Review			1. Harsh treatment during preparation of the oil reduces its quality.	1. Coconut oils are easily digestible and absorbable<br>2. Consumption of coconut oil increase the activities of enzymatic antioxidants such as catalase and superoxide dismutase which are involved in the detoxification of peroxide and superoxide radicals.<br>3. Efficiently consumption of coconut oil reduces acute inflammation.<br>4. Coconut oil reduces cholesterol level<br>5. Coconut oil reduces cardiovascular diseases<br>6. Coconut oil plays an important role in regulating insulin resistance and associated glucose metabolism.<br>7. Fermented VCO has shown to reduce paracetamol-induced toxicity by restoring liver function markers and hepatic morphology<br>8. It has been shown that fermented VCO possess antibacterial activities against a variety of strains including Candida
Abdullahi et al<br><br>2023<br><br>Nigeria	To provide an overview of quality variation and various contaminants in locally processed palm oil	Review			1. Poor handling and transportation lead to adulteration of the palm oil.<br>2. The variations in the chemical composition of the palm fruit, environmental factors, variation in processing leads to Microbial contamination. Higher microbial counts and the presence of toxic microorganisms.	1. Palm oil provides essential nutrients for growth

					3. Frequent consumption of contaminated oil leads to accumulation of metals in body fluids and vital organs.<br>4. Consumption of unsafe adulterated palm oil cause complicated health conditions that can lead to death.	
Kappally et al<br>2015<br>UAE	This article attempts to scientifically review the therapeutic benefits of this oil.	Review			1. The myth or perception of coconut oil being a deleterious to health as it would block the arteries and cause heart disease.	1. Palm oil is helpful in skin care, hair care, stress relief, weight loss and cholesterol level maintenance, immunomodulatory effects and cardiovascular uses.<br>2. Palm oil acts as anti-stress and antioxidant which reduces lipid peroxidation and increase the activity of SOD in the serum of mice undergoing the forced swim test and the brains of mice subjected to chronic cold restraint<br>3. Coconut oil as anti-inflammatory.<br>4. It aids in Wound healing as where the skin or other body tissue repairs itself after injury.<br>5. Coconut oil protects the skin from chronic skin diseases.<br>6. Coconut oil serves as an ocular rewetting agent.<br>7. Coconut oil prevents blood pressure.<br>8. Daily intake of coconut oil causes reduction in body fat accumulation.
Tan et al, 2021<br>Malaysia	This review is aimed to provide a comprehensive overview of the physicochemical properties and extraction processes of red palm oil, its nutritional properties and applications in food.	Review			1. Palm oil can generate reactive oxygen series that will induce DNA damage	2. Palm oil is an excellent source of carotenoids for pro-vitamin A.<br>3. Palm oil Increases serum retinol and lower cholesterol levels<br>4. Palm oil Increases antioxidant level and reduce risk of cancer
Nondzor et al, 2015<br>Ghana	To ascertain consumer's knowledge, perceptions and preference of edible oil(palm oil, coconut oil, sunflower oil, groundnut oil and palm kernel oil)	Cross sectional	Ghana<br>Males and females	206<br>183 females<br>23 males	1. Unrefined oils are high in cholesterol.	1. Edible oils have health benefits and nutritional values.

Koushki et al, 2015<br><br>Iran	To review the physicochemical properties, fatty acid profile and nutrition in palm oils.	Review			1. The composition of fatty acid of palm oil is assumed to be a cause of coronary heart disease.	1, Palm oil contains a high concentration of natural carotenoids<br>2. Palm oil prevents cancer<br>3. Palm oil has higher oxidative stability and results in high quality and tasty food.
	This systematic review focused on studies that reported the association between Virgin Coconut Oil and the biochemical measurements associated with cardiovascular diseases.	Review			1. High amount of consumption leads to overdose of vitamin E	1. Virgin Coconut Oil controls blood sugar and prevent high blood pressure.<br>2. Virgin Coconut Oil is rich in polyunsaturated acids which are beneficial to cardiovascular health.<br>3. Consumption of palm oil leads to reduction in obesity.<br>4. Virgin Coconut Oil improves the lipid profile by decreasing the concentrations of total cholesterol, triglycerides and low-density lipoprotein cholesterol
Deen et al, 2021<br><br>Sri Lanka	This review discusses the composition and functional properties of coconut oils extracted using various processing methods.	Review			1. Lack of supportive scientific evidence deters people from coconut oil consumption.	1. Coconut oil has hypocholesterolemia, anticancer, antihepatosteatotic, antidiabetic, antioxidant, anti-inflammatory, antimicrobial and skin moisturizing properties
Santos et al., 2019<br><br>Brazil	To determine coconut oil intake and its effects on the cardiometabolic profile	Review				1. Coconut oil has the following health benefits such as cardiovascular disease (CVD), weight loss, Alzheimer's disease, bone loss, diabetes, dental caries and prevention of atopic dermatitis and hair damage.
Joshi et al., 2020<br><br>India	To evaluate the antiviral, antibacterial and antifungal benefits of coconut oil.	Review				1. Coconut oil act as antioxidant and natural antibiotic and also help modulate immunity<br>2. Coconut oil helps in cholesterol lowering, reduction of cardiovascular disease risk, weight loss, appetite curb,<br>3. Coconut oil improves cognitive functions and strengthening of immune system.
Ntsefong et al., 2016	To review overview of controversies surrounding the oil palm production and consumption.	Review			1. High fat intake increases the risk of heart disease.	1. Palm oil is the main traditional cooking oil

Cameroon					2. Palm oil causes cancer due to formation of acrylamide at high frying temperatures	
Boateng et al., 2016<br><br>Ghana	The aim of this paper is thus to contest the negative publicity that coconut and palm oils have suffered, via exploring their unique potential roles in the nutritional and health status of the less endowed peoples of the world, (particularly those from the West African Subregion), and in improving food security to enhance national development	Review			1. Excessive intake of palm and coconut is strongly associated with an increased prevalence of obesity and an increased risk of developing coronary artery disease, high blood pressure, diabetes mellitus, and certain types of cancer.	1. Palm and coconut oils serves as a carrier of preformed fat-soluble vitamins, enhancing the bioavailability of fat-soluble micronutrients and providing essential substrate for the synthesis of metabolically active compounds<br>2. Palm and coconut oil have been used as a primary source of dietary fat as well as a remedy for illnesses<br>3. Palm oil contains carotenes which are precursors of vitamin A, which prevents night blindness, aids in maintenance of tissues and promotes growth.<br>4. Palm oil is rich in vitamin E, which is composed mainly of tocopherols and tocotrienols. Which acts as antioxidants reducing plasma cholesterol.
Vogel, et al., 2020<br><br>Brazil	To evaluate the influence of coconut oil on body composition, lipid profile 36 and glycemia in men with obesity	Randomized control clinical trial	Men with obesity	29		1. Coconut and soyabeans oil consumption reduces obesity<br>2. Coconut has effect on management of diabetes.
Jayawardena et al., 2021<br><br>India	To systematically summarized all high-quality evidence on the usage of coconut oil for health-related benefits.	Review				1. Coconut oil helps in the prevention and treatment of atopic dermatitis and oil pulling for the prevention of dental caries.<br>2. Coconut oil helps in cholesterol-lowering,<br>3. Coconut oil reduce the risk of cardiovascular diseases<br>4. Coconut oil aids in weight loss and improvement of cognitive functions,<br>5. Coconut oil consumption reduces risk of diabetes, and also enhance the immune system, and promoting wound healing

Konar et al., 2020<br><br>India	To find out the efficacy of coconut oil application for skin maturity, prevention of sepsis, hypothermia and apnea, its effect on long-term neurodevelopment and adverse effect of it.	Randomized control trial	Infants	2294		1. Use of coconut oil helps in dermal maturity and better neurodevelopmental outcome.<br>2. Smearing coconut oil on the new-borns reduces water loss and hypothermia thereby reducing infections and newborn mortality<br>3. Coconut oil is also helpful in promoting growth
Chew, 2018<br><br>Malaysia	To determine the benefit of coconut oil in management of atopic dermatitis	review				1. Virgin Coconut oil is useful in the management of Atopic Dermatitis<br>2. Coconut oil is use as antioxidant and anti-inflammatory<br>3. Coconut oil is useful in healing of wounds
